# Reconstruction of the acetabulum in THA using femoral head autografts in developmental dysplasia of the hip

**DOI:** 10.1186/1749-799X-6-32

**Published:** 2011-06-22

**Authors:** Markus D Schofer, Thomas Pressel, Jan Schmitt, Thomas J Heyse, Ulrich Boudriot

**Affiliations:** 1 Department of Orthopaedics and Rheumatology, University Hospital Marburg, Germany; 2 Department of Orthopaedic, Sankt Elisabeth Hospital, Gütersloh, Germany

**Keywords:** Bone graft, developmental dysplasia of the hip, primary total hip arthroplasty, THA

## Abstract

**Background:**

Severe acetabular deficiencies in cases of developmental dysplasia of the hip (DDH) often require complex reconstructive procedures in total hip arthroplasty (THA). The use of autologous femoral head grafts for acetabular reconstruction has been described, but few data is available about clinical results, the rates of non-union or aseptic loosening of acetabular components.

**Methods:**

In a retrospective approach, 101 patients with 118 THA requiring autologous femoral head grafts to the acetabulum because of DDH were included. Six patients had died, another 6 were lost to follow-up, and 104 hips were available for clinical and radiological evaluation at a mean of 68 ± 15 (13 to 159) months.

**Results:**

The average Merle d'Aubigné hip score improved from 9 to 16 points. Seven implants had to be revised due to aseptic loosening (6.7%). The revisions were performed 90 ± 34 (56 to 159) months after implantation. The other hips showed a stable position of the sockets without any signs of bony non-union, severe radiolucencies at the implant-graft interface or significant resorption of the graft.

**Conclusion:**

The use of autologous femoral head grafts with cementless cups in primary THA can achieve promising short- to midterm results in patients with dysplastic hips.

## Background

Stable and correct positioning of the socket in cases of developmental dysplasia of the hip (DDH) with subsequent severe bone stock deficiencies is one of the most challenging problems in total hip arthroplasty (THA). This is especially true in Crowe type II, III and IV hips [1]. While various shelf procedures have been used for operative treatment of DDH since the last century, Merle d'Aubigné [2] was the first to report on the reconstruction of the deficient acetabular roof, in cases of dysplastic hip joints using a Judet prosthesis and massive autologous bone grafts. This procedure was later improved in both primary and revision THA [3,4].

Detailed preoperative planning is needed in order to offer solutions which provide efficient bony support to restore the anatomic hip centre. The use of autologous and homologous bone grafts [5-19] as well as bone cement seals and reinforcement with metal rings or plates [20-26] have been described. Differing failure rates in the literature seem to depend on the follow-up time. However, the medium to long-term results of the different operative techniques remain contradictory. Autologous and homologous acetabular bone grafts were both reported to fail in the long-term due to non-union to the host bone and the subsequent mechanical failure, resulting in a breakdown of the bony structure of the transplanted bone followed by migration and loosening of the cup [9,27].

The purpose of the present study was to review the results of the treatment of severe acetabular deficiencies in DDH with autologous bone grafts in THA at the authors' institution. The hypotheses were that good short- to midterm results and a low complication rates can be achieved with this operative procedure.

## Methods

In a retrospective approach all THA cases in DDH requiring the use of autologous femoral head grafts at the acetabulum performed at the authors' institution in a 12-year period were identified from medical records. Full ethical approval was granted for the project by the local ethics committee. Informed consent was obtained in all cases prior to the inclusion into this study.

A contained acetabular defect was a necessary requirement for inclusion into the study. Acetabular discontinuity based on the radiological findings and intraoperative confirmation was evaluated. Femoral head grafts were indicated when > 20% of the cup remained uncovered by bone in its ideal position.

An anatomic cementless socket with a peg and a titanium mesh surface to facilitate bone ingrowth was used in all hips (Griss cup, Sulzer Medica, Switzerland) [28]. The acetabular components were implanted in a press fit technique and additionally fixed with nails. An effort was made to place the socket at the level of the original acetabulum. Autologous bone grafts from the harvested femoral head were used in all cases.

Autologous bone was always harvested at time of the index surgery and no sterilisation procedures or other additional processing were undertaken. Grafts were usually fixed to the lateral defect of the acetabulum with two cancellous compression screws and washers. The operative technique was originally described by Andrian-Werburg and Griss et al. [3]. Postoperative non-weight-bearing of the operated limb was necessary for 6 weeks. Physiotherapy was applied to mobilize the hip joint. Full weight bearing was allowed after three months.

The clinical results were analysed according to the Merle d'Aubigné hip score [29]. Antero-posterior (AP) radiographs of the hip were scanned and analysed with the DiagnostiX^®^-software system (Gemed, Freiburg, Germany). Radiolucencies at the bone-socket interface were classified using three zones as described by DeLee and Charnley [30]. Graft incorporation was assessed by the disappearance of the radiolucent line between graft and host bone and the remodelling of the inner structure of the bone graft. Coverage of the socket by bone graft was measured according to the DeLee/Charnley zones [30].

Reconstruction of the anatomic hip centre is an important part of any hip procedure. The centre of rotation of the hip joint can be determined in unilateral disease by mirroring the opposite, non-affected side. In the other cases, a previously described method was used to determine the anatomic rotation centre of the hip [31].

Wear of the polyethylene socket was measured on radiographs by determining the difference between the position of the femoral head inside the socket after index operation and at the latest follow-up. The radiographic measurement was made as described by Griffith et al. [32]. Clinical failure was defined as any need for revision of the acetabular component.

The measurements for the cups' individual movement directions were evaluated using a mixed linear model. The basis for this was the immediate postoperative image. For the observation of the change in position over the entire period, a variance analysis (F-test) was applied. All available radiographs were used for the adaptation of the model. The evaluation was carried out using the statistics programme "R" of the R-Foundation for Statistical Computing, Vienna, Austria. The Wilcoxon sign rank sum test was used to compare the Merle d'Aubigné hip scores. The significance level was set at p < 0.05.

## Results

A total of 101 patients (118 hips, 100 female, 18 male) could be identified. Six patients (6%) were lost to follow-up and another six patients died of reasons unrelated to surgery with implants still in place. 89 patients were available for clinical and radiological follow-up (104 hips) at an average postoperative follow-up of 68 ± 15 (13 to 159) months. This study group included 87 female hips and 17 male hips. The mean age at operation was 56 ± 11 (23 - 86) years and the average body mass index (BMI) was 26.4 ± 4.5 (17.8 – 50.1).

The Crowe classification for each hip dysplasia was determined preoperatively and showed type II in 41 cases, type III in 42 cases and type IV in 21 cases [1].

The postoperative Merle d'Aubigné score was 16.3 ± 2.1 points compared to 9.1 ± 1.4 points prior to operation. The postoperative improvement of an average of 6.5 ± 1.1 points was statistically significant (p < 0.01). The lateral inclination angle of the sockets was reduced from a preoperative 54.2 ± 10.7 to 38.2 ± 9.4 (range 16 - 62) degrees on average (p < 0.01).

**Figure 1:**
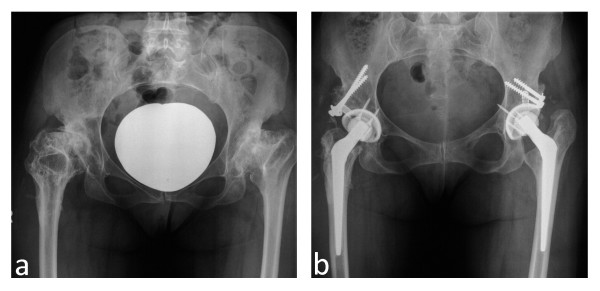
**a-b: Anteroposterior pelvic radiograph of a 43 year old female with bilateral hip dysplasia and coxarthrosis**. (a). Pelvic radiograph made five years after right and 6 years after left THA. The sockets are stable, and the bone grafts have healed (b).

There were no radiological signs of non-union or graft necrosis in the included cases (Figure 1). All grafts were incorporated within twelve months after operation judging by serial radiographs. Resorption of lateral parts of the bone graft was considered significant only if it exceeded the lateral unloaded rim of the socket. Four such cases were seen but the resorption was restricted only to the lateral edge of the graft. The bone coverage of the socket was not affected and all implants appeared radiologically and clinically stable.

**Figure 2:**
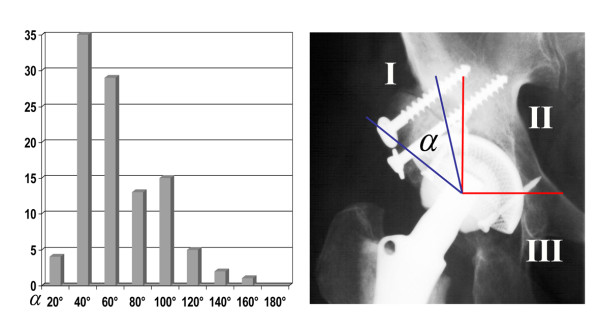
**Measurement of socket/graft coverage according to Charnley and DeLee; α = coverage angle**.

According to the DeLee/Charnley zones, the coverage of the socket by the bone graft was measured. 78% covered zone I, 19% zone I and II and 3% zone all three zones (mean: 57 degrees of a possible maximum of 180 degrees) (Figure 2).

**Figure 3:**
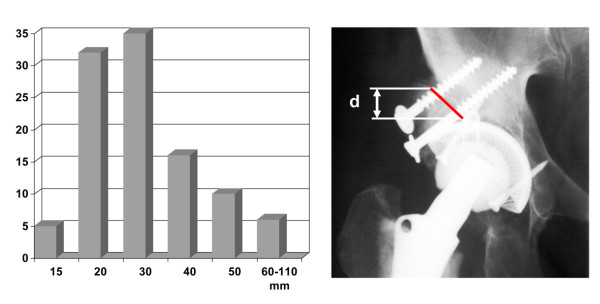
**Measurement of host/graft contact area**.

**Figure 4:**
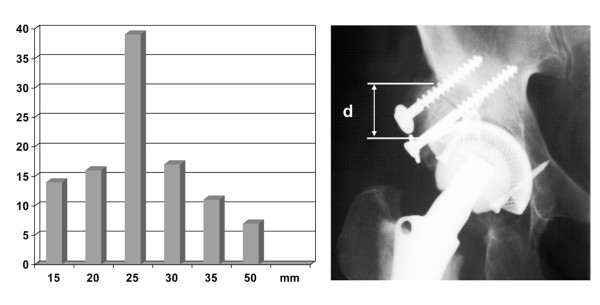
**Graphic representation of measurements of the autologous graft thickness**.

The length of the contact zone between graft and host bone was in mean 36 ± 4 mm (range 12 to 110 mm), when measured on AP radiographs (Figure 3). The graft thickness ranged from 1 - 5 cm with a maximum of 2.6 cm for both autologous and homologous grafts (Figure 4).

In congenital hip dysplasia it has to be considered that the hip centre is often located relatively high. A correction of the hip centre by 20.8 mm into the medial and 11.4 mm into the distal direction on average could be shown (p < 0.01). Wear of the polyethylene socket was 1.2 ± 0.4 mm at the latest follow-up.

Complications after operation occurred in 16 cases: one deep vein thrombosis, four femoral nerve palsies, and three patients suffered a dislocation of the hip. Heterotopic ossifications occurred in eight cases. All these cases were operated in the time before routine prophylaxis with Indometacine or low-dose irradiation was introduced as a standard procedure at the authors' institution. No infections were seen.

Seven patients were revised for aseptic loosening of the socket. The revisions were carried out within 56 to 159 months after implantation (90 ± 34 months). In all revision cases, the transplanted grafts were intraoperatively seen to be vital. The grafts were evaluated macroscopically and had normal bleeding characteristics after drilling and reaming. In two cup revision cases, a cementless pressfit socket was used, in two other cases a cemented socket and in three cases, an acetabular cage with a cemented cup was applied.

Seven of all operated hips showed radiolucent lines at the socket-graft interface, which were all less than two millimetres in thickness at latest review. Two of these were in DeLee and Charnley zone I, 1 in zone II, 2 in zones I and II and 2 in zones II and III. These cases were not considered a failure.

Migration of the socket was seen in six cases 12 to 58 months after surgery (31 ± 21 months). However, there was no clinical evidence of loosening of the socket in these cases and an annual radiological examination was recommended.

## Discussion

The presented data show that severe bony defects due to DDH can be successfully reconstructed biologically. The use of autologous femoral head grafts with cementless cups in primary THA can achieve promising short- to midterm results in patients with hip dysplasia.

There are some limitations to this study mainly due to its retrospective design and the follow-up range from 13 to 159 months. To estimate radiolucencies and signs of socket loosening, serial X-rays were analyzed. However, the extent of radiolucent lines and tilting or subsidence of the cup remains difficult to assess. Results of x-ray examinations should be analysed with caution. Variation of the pelvic position between radiographs may lead to a change of at least five degrees or two to three millimeters in cup position or thickness of radiolucencies. There are limitations in ensuring graft integration by plain radiographs. In revision cases with cup loosening graft vitality was evaluated macroscopically. No histological analysis of biopsies was performed.

There is no doubt about the need to restore the anatomic hip centre and provide a good initial and long-term stability in cases of severe acetabular deficiency due to congenital hip dysplasia especially in Crowe type II, III and IV hips [1]. There are several methods to achieve this goal. However, Morand et al. [33] reported a failure rate of 13% with an average follow-up of 7.3 years using bulk allografts and cemented cups. McCollum et al. [13], Marti et al. [34] and Hartwig et al. [35] reported similar results. Stans et al. [36] found 53% loose cemented acetabular components at an average of 16.6 years. They pointed out that the reconstruction of the femoral head centre is predictive of successful long term acetabular component fixation. The loosening rate rose up to 83.3% in cases of cup positioning outside the anatomic hip centre. However, bulky cement seals were used to fill large bone defects which could explain these unsatisfactory results.

In our experience, there are a number of factors that influence the successful incorporation of autologous massive grafts:

1. Quality of bone: Femoral heads retrieved from cases of primary DDH are mechanically more stable than homologous grafts taken from patients with femoral neck fractures with a high likelihood of osteoporosis.

2. Graft orientation in relation to the host bone is of utmost importance. We always try to bring the subchondral sclerotic part of the graft in contact with the sclerosis of the acetabular roof and the loaded area of the new socket. Thus, the graft is always inserted as an inlay and not as an onlay graft [4].

3. Screw orientation is also of significance. We recommend screw orientation for graft fixation close or parallel to the ideal resultant hip force. Horizontal or close to horizontal screw placement increases screw fracture and graft resorption or migration. Axial compression of the graft and the reconstructed acetabular roof by correct screw placement enhances bone remodelling and graft incorporation.

4. The reconstruction of the anatomical rotational centre of the hip is of particular importance [37,38]. So the restoration of a physiological load transfer from the socket through the graft to host bone gives the most favourable basis for incorporation and remodelling of the graft.

5. Matching of defect and graft size and shape is often technically demanding but essential for primary stability of the construction and successful incorporation of the graft under load. In primary THA, the femoral head is therefore fixed to the acetabular defect „face to face". Then reaming is started medially into the graft.

6. The selection of socket design for non cemented implantation is also of importance. In our early experience in the 70's screw-in sockets or square-shaped sockets [39,40] proved to be less successful, supposedly mainly due to design and the material used at this time. We now prefer anatomical press-fit sockets. If there are problems with graft stability or graft fitting, acetabular supporting shells with cemented cups should be given preference.

With these considerations it seems to be difficult to compare the presented results with those of other authors.

Reports of revision operations with histological evidence of osteonecrosis of the graft and only partial or no graft incorporation may reflect rather technical problems of graft fixation than the general biological fate of both homologous and autologous grafts. The higher failure rate of massive homologous grafts in other series [7,9,34,41,42] can not only be attributed to the nature of homologous grafts alone but at least in part also to the poorer bone quality and regenerative capacity of the host bone in revision cases. Exact fitting of the graft, screw placement and tight fixation in arthroplasties can be quite difficult in highly deficient acetabula, especially in older patients whereas bone quality in primary THA for severe acetabular dysplasia is usually good and the patient's are younger.

It has been suspected that the remodelling process cannot reach the inner core of massive structural bone grafts. In this respect, autologous and homologous grafts have to be discussed separately. Marti et al. [34] preferred an operative technique of reconstructing the deficient acetabulum using bulk autologous grafts harvested from the iliac bone of the patient or in the case of primary THA grafts from the femoral head. Bulk grafts were cut into two or three smaller pieces to facilitate revascularisation and were attached with screws or plates. In all cases, osteointegration of the graft was seen. In the case of homologous grafts, the results seem to be worse. Histological findings showed no remodelling of the central part of the transplanted homologous bone samples [43,44]. Apparently a bulk homologous graft is able to provide long-term stability despite incomplete remodelling of the core. On the other hand, Gordon et al. [45] demonstrated by single photon emission computed tomography (SPECT) analysis normal radionuclide activity as a sign of osseointegration for both autologous and homologous femoral head grafts four to seven years after the operation. Positron emission tomography (PET) can be used to study metabolic events in vivo. Ullmark et al. analyzed the course of bone healing in the impacted allograft beds in the acetabulum using PET [46].

Assuming that the remodelling process depends on the blood supply of the graft, it is necessary to direct attention to an improved operative technique. To what extent the revascularisation can be accelerated by small drill holes into the graft is matter of discussion.

To improve bone remodelling some authors favours the use of cortico-cancellous bone chips. Good results with this method were reported by Azuma et al. [47] and Heekin et al. [48]. However, it remains questionable if this method is useful in cases of uncontained defects, when initial stability cannot be achieved. To avoid an initial instability and to protect the graft, the use of metal supporting rings is proposed [22,49]. In cases of severe forms of congenital hip dysplasia, the reconstruction of the deficient lateral rim of the acetabulum with morsellised cancellous bone chips as well as stabilisation with screw or press-fit sockets appears difficult or impossible to perform.

During revision surgeries performed in this series a substantial incorporation of the autologous graft was observed in all cases. Thus, precise reaming and placement of a new socket was facilitated in the revision procedure. Bal et al. [50] found good clinical and radiological results after at 76 months follow-up after revision THA using the previous transplanted bulk femoral head grafts as bone stock for the support of the new cementless socket.

Differing failure rates in the literature also seem to depend on the follow-up time. Mulroy and Harris [27] emphasize that a late failure of bulk allograft is to be expected. They found a total of 46% of loose cups after a mean follow up of 11.8 years. Five years earlier, all sockets seemed to be stable. A longer follow-up of the presented series will show if the yet promising results can be confirmed. So far, a failure rate of 6.7% is encouraging.

## Conclusions

In conclusion, the use of autologous femoral head grafts with cementless cups in primary THA can achieve promising short- to midterm results in patients with hip dysplasia.

## List of abbreviations

AP: Anteroposterior; BMI: Body mass index; DDH: Developmental Dysplasia of the Hip; PET: Positron emission tomography; SPECT: Single photon emission computed tomography; THA: Total Hip Arthroplasty.

## Competing interests

The authors declare that they have no competing interests.

## Authors' contributions

MDS drafting of the manuscript, analysis and interpretation of data, revision and final approval of manuscript

TP acquisition of data, analysis and interpretation of data, revision and final approval of manuscript

JS acquisition of data, analysis and interpretation of data, revision and final approval of manuscript

TJH analysis and interpretation of data, drafting of the manuscript, revision and final approval of manuscript

UB conception and design of the study, revision and final approval of manuscript

## References

[B1] Crowe JF Mani VJ Ranawat CS Total hip replacement in congenital dislocation and dysplasia of the hip J Bone Joint Surg Am 1979 61 1 15 23 365863

[B2] Merle D'Aubigne RM Reposition with arthroplasty for congenital dislocation of the hip in adults J Bone Joint Surg Br 1952 34-B 1 22 29 12999865 10.1302/0301-620X.34B1.22

[B3] Griss P Jentschura G Heimke G [A new technique for socket implantation into dysplastic acetabula (author's transl)] Arch Orthop Trauma Surg 1978 93 1 57 63 10.1007/BF00386552 727931

[B4] Harris WH Crothers O Oh I Total hip replacement and femoral-head bone-grafting for severe acetabular deficiency in adults J Bone Joint Surg Am 1977 59 6 752 759 908698

[B5] Avci S Connors N Petty W 2- to 10-year follow-up study of acetabular revisions using allograft bone to repair bone defects J Arthroplasty 1998 13 1 61 69 10.1016/S0883-5403(98)90076-6 9493539

[B6] Emerson RH Jr Head WC Berklacich FM Malinin TI Noncemented acetabular revision arthroplasty using allograft bone Clin Orthop Relat Res 1989 249 30 43 2582676

[B7] Garbuz D Morsi E Gross AE Revision of the acetabular component of a total hip arthroplasty with a massive structural allograft. Study with a minimum five-year follow-up J Bone Joint Surg Am 1996 78 5 693 697 8642025 10.2106/00004623-199605000-00008

[B8] Gerber SD Harris WH Femoral head autografting to augment acetabular deficiency in patients requiring total hip replacement. A minimum five-year and an average seven-year follow-up study J Bone Joint Surg Am 1986 68 8 1241 1248 3771605

[B9] Hooten JP Jr Engh CA Jr Engh CA Failure of structural acetabular allografts in cementless revision hip arthroplasty J Bone Joint Surg Br 1994 76 3 419 422 8175845

[B10] Inao S Gotoh E Ando M Total hip replacement using femoral neck bone to graft the dysplastic acetabulum. Follow-up study of 18 patients with old congenital dislocation of the hip J Bone Joint Surg Br 1994 76 5 735 739 8083261

[B11] Jasty M Harris WH Salvage total hip reconstruction in patients with major acetabular bone deficiency using structural femoral head allografts J Bone Joint Surg Br 1990 72 1 63 67 2298796 10.1302/0301-620X.72B1.2298796

[B12] Lachiewicz PF Hussamy OD Revision of the acetabulum without cement with use of the Harris-Galante porous-coated implant. Two to eight-year results J Bone Joint Surg Am 1994 76 12 1834 1839 7989389 10.2106/00004623-199412000-00010

[B13] McCollum DE Nunley JA Harrelson JM Bone-grafting in total hip replacement for acetabular protrusion J Bone Joint Surg Am 1980 62 7 1065 1073 7430192

[B14] McGann WA Welch RB Picetti GD Acetabular preparation in cementless revision total hip arthroplasty Clin Orthop Relat Res 1988 235 35 46 3416540

[B15] Paprosky WG Magnus RE Principles of bone grafting in revision total hip arthroplasty. Acetabular technique Clin Orthop Relat Res 1994 298 147 155 8118969

[B16] Samuelson KM Freeman MA Levack B Rassmussen GL Revell PA Homograft bone in revision acetabular arthroplasty. A clinical and radiographic study J Bone Joint Surg Br 1988 70 3 367 372 3286655 10.1302/0301-620X.70B3.3286655

[B17] Slooff TJ Buma P Schreurs BW Schimmel JW Huiskes R Gardeniers J Acetabular and femoral reconstruction with impacted graft and cement Clin Orthop Relat Res 1996 324 108 115 10.1097/00003086-199603000-00013 8595745

[B18] Trancik TM Stulberg BN Wilde AH Feiglin DH Allograft reconstruction of the acetabulum during revision total hip arthroplasty. Clinical, radiographic, and scintigraphic assessment of the results J Bone Joint Surg Am 1986 68 4 527 533 3485633

[B19] Schreurs BW Keurentjes JC Gardeniers JW Verdonschot N Slooff TJ Veth RP Acetabular revision with impacted morsellised cancellous bone grafting and a cemented acetabular component: a 20- to 25-year follow-up J Bone Joint Surg Br 2009 91 9 1148 1153 10.1302/0301-620X.91B9.21750 19721038

[B20] Berry DJ Muller ME Revision arthroplasty using an anti-protrusio cage for massive acetabular bone deficiency J Bone Joint Surg Br 1992 74 5 711 715 1527119 10.1302/0301-620X.74B5.1527119

[B21] Gill TJ Sledge JB Muller ME The management of severe acetabular bone loss using structural allograft and acetabular reinforcement devices J Arthroplasty 2000 15 1 1 7 10.1016/S0883-5403(00)90973-2 10654455

[B22] Gurtner P Aebi M Ganz R [The acetabular roof cup in revision arthroplasty of the hip] Z Orthop Ihre Grenzgeb 1993 131 6 594 600 10.1055/s-2008-1040077 8310753

[B23] Massin P Tanaka C Huten D Duparc J [Treatment of aseptic acetabular loosening by reconstruction combining bone graft and Muller ring. Actuarial analysis over 11 years] Rev Chir Orthop Reparatrice Appar Mot 1998 84 1 51 60 9775022

[B24] Rosson J Schatzker J The use of reinforcement rings to reconstruct deficient acetabula J Bone Joint Surg Br 1992 74 5 716 720 1527120 10.1302/0301-620X.74B5.1527120

[B25] Ochs BG Schmid U Rieth J Ateschrang A Weise K Ochs U Acetabular bone reconstruction in revision arthroplasty: a comparison of freeze-dried, irradiated and chemically-treated allograft vitalised with autologous marrow versus frozen non-irradiated allograft J Bone Joint Surg Br 2008 90 9 1164 1171 10.1302/0301-620X.90B9.20425 18757955

[B26] Kawanabe K Akiyama H Onishi E Nakamura T Revision total hip replacement using the Kerboull acetabular reinforcement device with morsellised or bulk graft: results at a mean follow-up of 8.7 years J Bone Joint Surg Br 2007 89 1 26 31 10.1302/0301-620X.89B1.18037 17259411

[B27] Mulroy RD Jr Harris WH Failure of acetabular autogenous grafts in total hip arthroplasty. Increasing incidence: a follow-up note J Bone Joint Surg Am 1990 72 10 1536 1540 2254363

[B28] Hinrichs F Boudriot U Held T Griss P [10 years results with a Monobloc hip endoprosthesis cup with multilayer titanium mesh coating for cement-free implantation] Z Orthop Ihre Grenzgeb 2001 139 3 212 216 10.1055/s-2001-16323 11486623

[B29] D'Aubigne RM Postel M Function al results of hip arthroplasty with acrylic prosthesis J Bone Joint Surg Am 1954 36-A 3 451 475 13163078

[B30] DeLee JG Charnley J Radiological demarcation of cemented sockets in total hip replacement Clin Orthop Relat Res 1976 121 20 32 991504

[B31] Boudriot U Hilgert J Hinrichs F Determination of the rotational center of the hip Arch Orthop Trauma Surg 2006 126 6 417 420 10.1007/s00402-006-0157-y 16758229

[B32] Griffith MJ Seidenstein MK Williams D Charnley J Socket wear in Charnley low friction arthroplasty of the hip Clin Orthop Relat Res 1978 137 37 47 743841

[B33] Morand F Clarac JP Gayet LE Pries P [Acetabular reconstruction using bone allograft in the revision of total hip prosthesis] Rev Chir Orthop Reparatrice Appar Mot 1998 84 2 154 161 9775059

[B34] Marti RK Schuller HM van Steijn MJ Superolateral bone grafting for acetabular deficiency in primary total hip replacement and revision J Bone Joint Surg Br 1994 76 5 728 734 8083260

[B35] Hartwig CH Beele B Kusswetter W Femoral head bone grafting for reconstruction of the acetabular wall in dysplastic hip replacement Arch Orthop Trauma Surg 1995 114 5 269 273 10.1007/BF00452085 7577218

[B36] Stans AA Pagnano MW Shaughnessy WJ Hanssen AD Results of total hip arthroplasty for Crowe Type III developmental hip dysplasia Clin Orthop Relat Res 1998 348 149 157 9553547

[B37] Pagnano W Hanssen AD Lewallen DG Shaughnessy WJ The effect of superior placement of the acetabular component on the rate of loosening after total hip arthroplasty J Bone Joint Surg Am 1996 78 7 1004 1014 8698717 10.2106/00004623-199607000-00004

[B38] Yoder SA Brand RA Pedersen DR O'Gorman TW Total hip acetabular component position affects component loosening rates Clin Orthop Relat Res 1988 228 79 87 3342591

[B39] Griss P Heimke G Five years experience with ceramic-metal-composite hip endoprostheses. I. clinical evaluation Arch Orthop Trauma Surg 1981 98 3 157 164 10.1007/BF00632972 7259461

[B40] Griss P Werner E Buchinger R Heimke G [The Mannheimer oxide ceramic-metal composite hip prostheses (author's transl)] Arch Orthop Unfallchir 1977 87 1 73 84 10.1007/BF00416141 836223

[B41] Callaghan JJ Salvati EA Pellicci PM Wilson PD Jr Ranawat CS Results of revision for mechanical failure after cemented total hip replacement, 1979 to 1982. A two to five-year follow-up J Bone Joint Surg Am 1985 67 7 1074 1085 4030826

[B42] Gross AE Hutchison CR Alexeeff M Mahomed N Leitch K Morsi E Proximal femoral allografts for reconstruction of bone stock in revision arthroplasty of the hip Clin Orthop Relat Res 1995 319 151 158 7554624

[B43] Enneking WF Mindell ER Observations on massive retrieved human allografts J Bone Joint Surg Am 1991 73 8 1123 1142 1890115

[B44] Hooten JP Jr Engh CA Heekin RD Vinh TN Structural bulk allografts in acetabular reconstruction. Analysis of two grafts retrieved at post-mortem J Bone Joint Surg Br 1996 78 2 270 275 8666640

[B45] Gordon SL Binkert BL Rashkoff ES Britt AR Esser PD Stinchfield FE Assessment of bone grafts used for acetabular augmentation in total hip arthroplasty. A study using roentgenograms and bone scintigraphy Clin Orthop Relat Res 1985 201 18 25 3905128

[B46] Ullmark G Sorensen J Nilsson O Bone healing of severe acetabular defects after revision arthroplasty Acta Orthop 2009 80 2 179 183 10.3109/17453670902947416 19404799 PMC2823168

[B47] Azuma T Yasuda H Okagaki K Sakai K Compressed allograft chips for acetabular reconstruction in revision hip arthroplasty J Bone Joint Surg Br 1994 76 5 740 744 8083262

[B48] Heekin RD Engh CA Vinh T Morselized allograft in acetabular reconstruction. A postmortem retrieval analysis Clin Orthop Relat Res 1995 319 184 190 7554629

[B49] Sadri H Pfander G Siebenrock KA Tannast M Koch P Fujita H Ballmer P Ganz R Acetabular reinforcement ring in primary total hip arthroplasty: a minimum 10-year follow-up Arch Orthop Trauma Surg 2008 128 8 869 877 10.1007/s00402-008-0612-z 18347806

[B50] Bal BS Maurer T Harris WH Revision of the acetabular component without cement after a previous acetabular reconstruction with use of a bulk femoral head graft in patients who had congenital dislocation or dysplasia. A follow-up note J Bone Joint Surg Am 1999 81 12 1703 1706 10608381 10.2106/00004623-199912000-00007

